# Lenalidomide-induced type II Kounis syndrome: a case report

**DOI:** 10.3389/fcvm.2025.1591179

**Published:** 2025-06-16

**Authors:** Ruihan Hu, Guoxiang He

**Affiliations:** Department of Cardiovascular Medicine, Guiqian International General Hospital, Guiyang, Guizhou Province, China

**Keywords:** myocardial infarction, Kounis syndrome, lenalidomide, allergy, interventional treatment

## Abstract

Kounis syndrome is an allergic myocardial ischemia syndrome triggered by various factors. Herein, we presented a case of a patient with Multiple Myeloma who developed chest pain as a result of lenalidomide use. The patient’s electrocardiogram (ECG) revealed ST-segment depression, along with elevated troponin I and eosinophils levels. Emergency coronary angiography identified thrombosis in the middle segment of the anterior descending branch of the left coronary artery. Previous case reports have linked lenalidomide chemotherapy to myocarditis as the primary form of myocardial damage. This case marks the first documented occurrence of myocardial infarction attributed to lenalidomide, highlighting a previously unrecognized aspect of its cardiotoxic profile.

## Case description

A 68-year-old male presented to the Orthopedic Department with chief complaints of right shoulder and back pain. The patient has been definitively diagnosed with the following conditions: (1) solitary skeletal plasma cell tumor, for which he received radiation therapy targeting the L2 lumbar vertebra (GTV = 50 Gy/25f). (2) Coronary atherosclerotic heart disease, managed with stent implantation in the left anterior descending artery and circumflex artery 10 years ago, and received long-term oral aspirin 100 mg once daily with uninterrupted antiplatelet therapy. (3) Type 2 diabetes mellitus. (4) Hypertension. The SPECT/CT fused image revealed the presence of multiple myeloma prior to the patient’s admission. He was subsequently transferred to the Hematology Department for chemotherapy. The patient was definitively diagnosed with International Staging System (ISS) stage I IgG Kappa-type Multiple Myeloma. Given the patient’s underlying cardiovascular disease, the chemotherapy regimen was determined to include lenalidomide 25 mg and dexamethasone 20 mg administered orally once daily in the morning.

**Figure 1:**
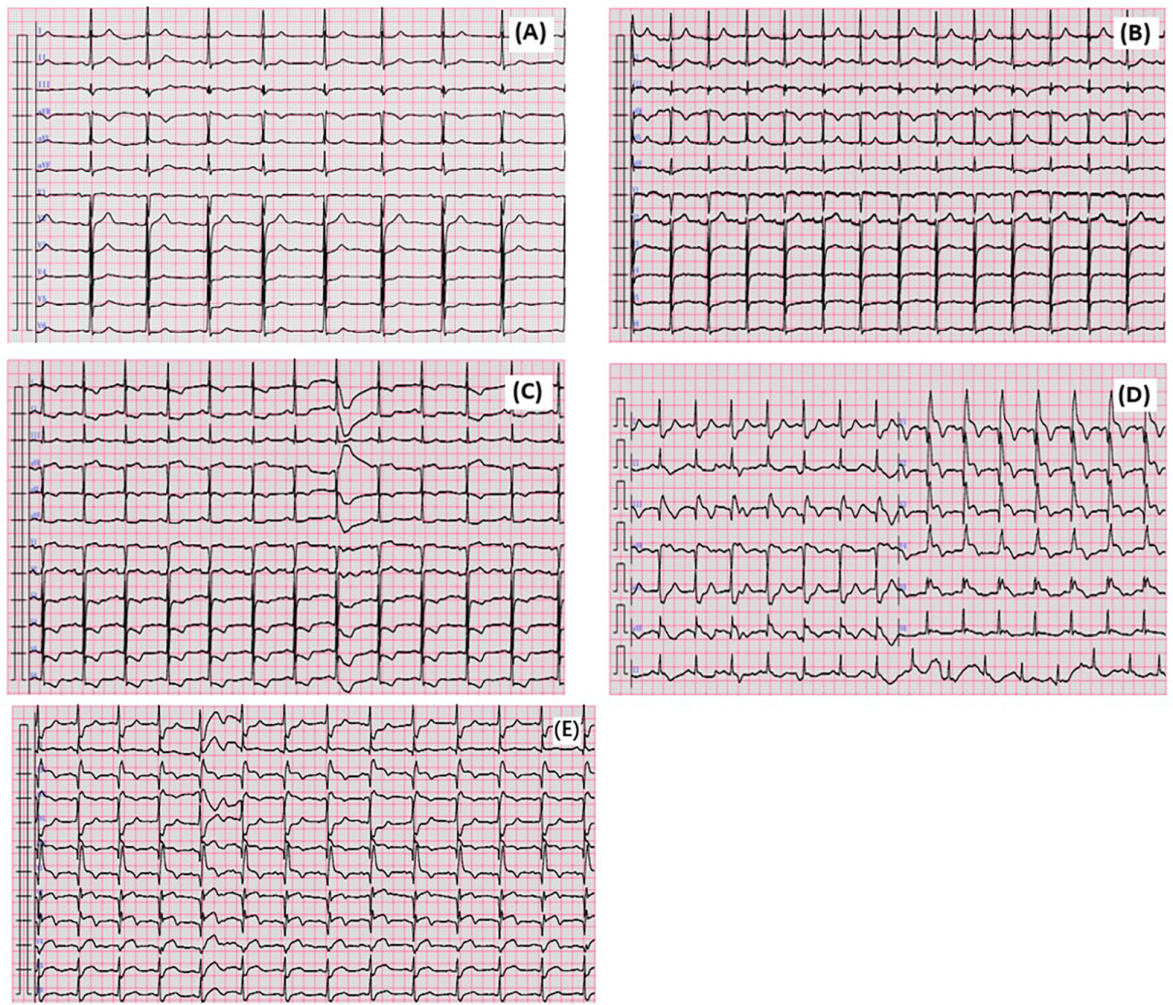
The ECG changed in this case. **(A)** Admission ECG. **(B)** Taking lenalidomide for 40 min ECG. **(C)** Taking lenalidomide for 14 h and 16 min ECG. **(D)** ECG after PTCA. **(E)** ECG after PTCA 24 h.

**Figure 2:**
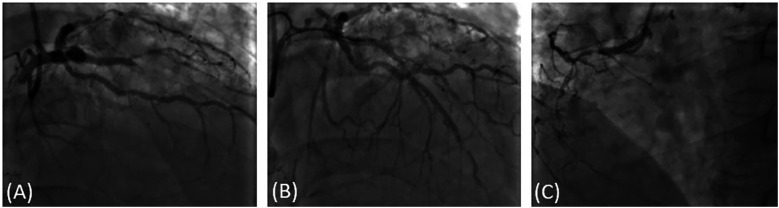
Coronary angiography image. **(A)** LAD and LCX, thrombosis formation in the anterior descending artery stent, the stent was completely obstructed. 50% stenosis in the proximal segment of LCX, proximal segment of RCA occlusion. **(B)** percutaneous coronary angioplasty in LAD, coronary flow recovery. **(C)** proximal segment of RCA occlusion.

The patient experienced mild chest tightness and pain for the first time 40 min after taking lenalidomide, with symptoms persisting for about 10 min before self-relief. 6 h and 30 min after medication, the patient experienced a second episode of chest pain, described as piercing pain, accompanied by shortness of breath, chest tightness, and itching skin. The ECG findings at this stage showed flattening of the T wave in leads V5 and V6 compared to that of the pre-symptomatic state (Figures 1A,B). Troponin I was <0.025 ng/ml (baseline level <0.025 ng/ml). The symptoms improved with sublingual administration of 0.5 mg nitroglycerin. After 14 h and 16 min of medication, the patient experienced a third episode of chest pain, which progressively worsened. The ECG demonstrated ST-segment depression in leads V4–V6, along with T wave inversion in V1–V6 leads (Figure 1C). Troponin I increased to 0.042 ng/ml, prompting transfer to the Cardiology Department. Given the patient’s poor baseline condition, the patient’s family initially declined immediate coronary angiography due to procedural risk. However, after 21 h and 25 min, troponin I further increased to 0.286 ng/ml, and the eosinophil count elevated to 3.42 × 10^9^/L. At this point, the family consented to surgery. The patient received Plavix 300 mg, and emergency coronary angiography revealed that: (1) complete thrombotic occlusion of the stent in the anterior descending artery; (2) 50% stenosis in the proximal segment of the left circumflex artery (LCX); (3) proximal segment of right coronary artery (RCA) occlusion; (4) percutaneous coronary angioplasty was performed in the LAD (Figure 2). Postoperatively, ECG demonstrated complete right bundle branch block and ST-segment elevation of 2–4 mm in leads III, aVR, aVF and V1–V5, with ST-segment depression in leads I and aVL (Figure 1D). The patient’s chest pain was relieved, and troponin I peaked earlier than expected, with ECG after 24 h showed a reduction in ST-segment elevation compared to immediate post-procedure levels (Figure 1E). Lenalidomide was discontinued, and the patient experienced no further episodes of chest pain. The patient was finally diagnosed as acute non-ST-segment elevation myocardial infarction due to allergic coronary vasospasm, type II Kounis syndrome, with lenalidomide identified as the triggering allergen.

**Figure 3:**
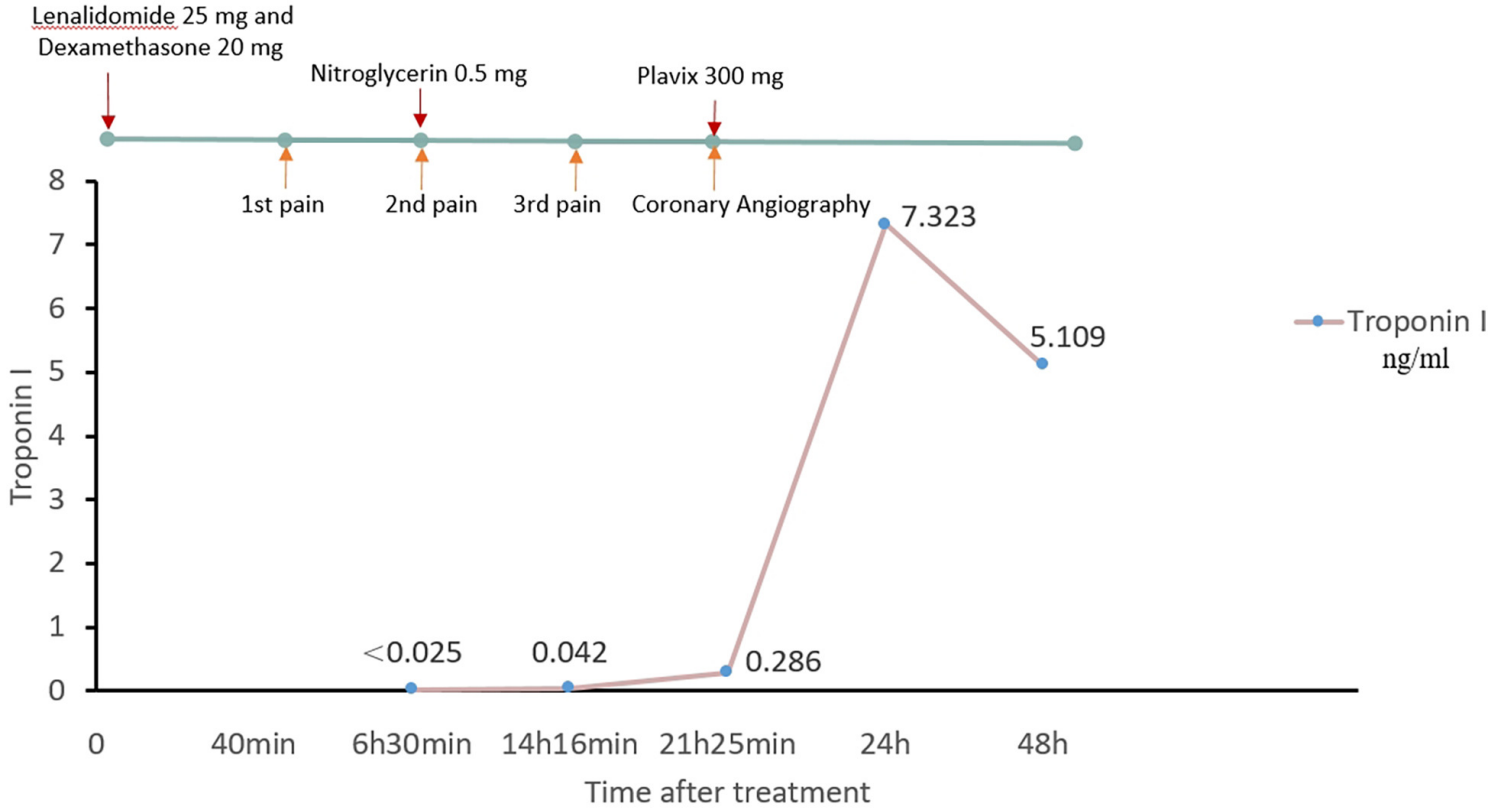
Time table.

## Discussion

Lenalidomide is an analog of thalidomide, exhibiting immunomodulatory, anti-angiogenic, and anti-tumor effects. Its immunomodulatory effects include increasing the number and activity of T-cells and natural killer cells, directly enhancing antibody-dependent cell-mediated cytotoxicity, and suppressing the release of pro-inflammatory cytokines (TNF-α and IL-6) from monocytes by stimulating IL-2 and IFN-γ secretion (1). The peak plasma concentration of lenalidomide occurs within 0.5–1 h post administration. The most common adverse reactions include thrombocytopenia and neutropenia. Other adverse reactions include diarrhea, itching, rash, fatigue, constipation, nausea, nasopharyngitis, joint pain, fever, back pain, peripheral edema, cough, dizziness, headache, muscle spasms, dyspnea, and pharyngitis (2). The instructions for lenalidomide list several special warnings, including the potential to induce birth defects and an increased risk of deep vein thrombosis and pulmonary embolism. In this case, the patient’s clinical deterioration was associated with coronary artery thrombosis induced by lenalidomide.

Kounis syndrome is a form of unstable angina or myocardial infarction triggered by allergic reactions. It was first identified and named by Kounis and Zavras in 1991 (3). The underlying mechanism involves allergens triggering allergic reactions upon entering the body. These reactions, combined with specific IgE antibodies, leads to cell activation and the release of inflammatory mediators. These mediators can induce coronary artery spasm, plaque erosion, rupture, and thrombosis within coronary stents. In a review published in 2016, Nicholas G. Kounis listed multiple discovered allergic factors that can cause Kounis syndrome, including analgesics, anesthetics, antibiotics, anticoagulants, anti-tumor drugs, contrast agents, glucocorticoids, nonsteroidal anti-inflammatory drugs, food, environment, etc. (4). Subsequently, new case reports of Kounis syndrome caused by Flurbiprofen, Chinese herbs, and Ibuprofen were discovered (5–7). Common allergens include medications, foods, and environmental factors. Kounis syndrome is categorized into three types: Type Ⅰ involves acute allergic reactions causing coronary spasm. Typically, patients do not exhibit coronary artery disease, with normal myocardial enzymes and troponin levels; Type Ⅱ involves acute allergic reactions triggering thrombosis, plaque erosion or rupture in patients with pre-existing coronary atherosclerotic disease, leading to acute myocardial infarction; Type III is an allergic reaction to coronary stent implantation, characterized by the release of the stent during surgery, resulting in thrombus formation inside the stent (8). Although Kounis syndrome is not rare, it is frequently misdiagnosed or misinterpreted due to its overlap with other cardiovascular conditions.

Currently, researchers suggest that investigating the source of allergens and personal medical history is crucial in diagnosing Kounis syndrome. Approximately 25.1% of patients have a history of allergies, and typically, allergens need to be traced back 1–6 h prior to clinical onset. In this case, the patient initially experienced chest pain 40 min after taking lenalidomide. Naranjo’s score for this case is 7 points, demonstrating the correlation between the drug and Kounis syndrome. Following the onset, dynamic evolution in the ECG indicated that the culprit vessel was located in the LAD. Coronary angiography confirmed this inference, revealing evidence of thrombosis in the patient, consistent with the characteristics of type Ⅱ Kounis syndrome. Previously, three cases of lenalidomide-induced myocarditis with ST-segment elevation have been reported (9–11). However, this case represents the first documented instance of Kounis syndrome caused by a lenalidomide allergy.

## Data Availability

The original contributions presented in the study are included in the article/Supplementary Material, further inquiries can be directed to the corresponding author.
